# Supplementary material for: On the mechanisms of lysis triggered by perturbations of bacterial cell wall biosynthesis
Source: Nat Commun. 2023 Jul 11;14:4123. doi: 10.1038/s41467-023-39723-8 (PMC10336097; doi:10.1038/s41467-023-39723-8)
Supplement: Supplementary file 1 — Supplementary Information [file 41467_2023_39723_MOESM1_ESM.pdf]

SUPPLEMENTARY INFORMATION for:

On the mechanisms of lysis triggered by perturbations of bacterial cell wall biosynthesis

Yoshikazu Kawai*, Maki Kawai, Eilidh Sohini Mackenzie, Yousef Dashti, Bernhard Kepplinger, Kevin John Waldron, Jeff Errington*

*Corresponding authors

yoshikazu.kawai@sydney.edu.au

jeffery.errington@sydney.edu.au

Contents:

Supplementary Figs. 1-7, Supplementary Table 1 and 2 and Supplementary References.

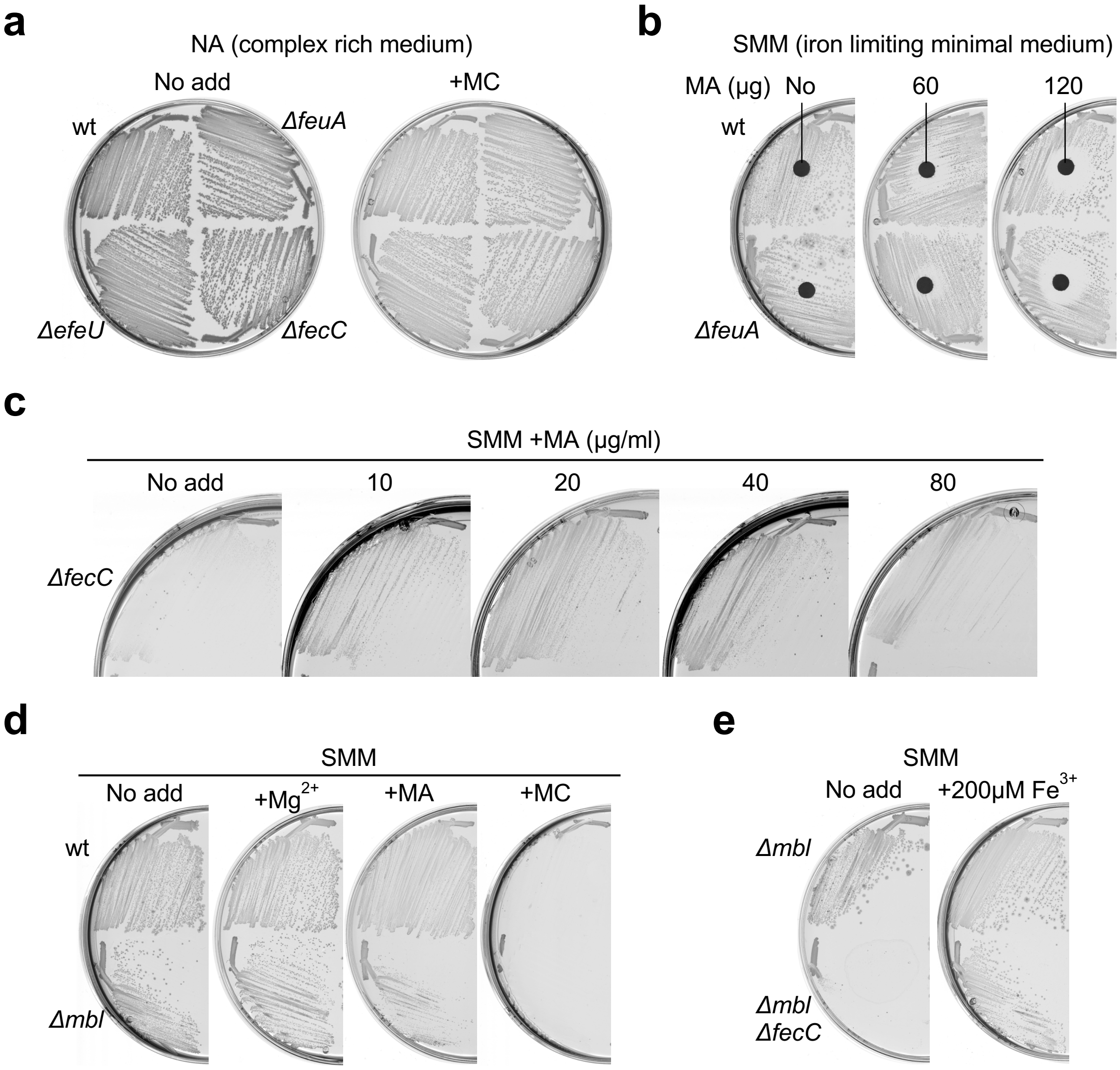

Supplementary Fig. 1. **Effects of Mirubactins and iron availability on *B. subtilis* growth.** **a** Effects of MC on *B. subtilis* growth in a complex medium. *B. subtilis* strains 168CA (wild-type), YK2739 (*ΔefeU*), YK2740 (*ΔfecC*) and YK2741 (*ΔfeuA*) were streaked on NA plates with or without 10 μg/ml MC, and incubated for 18 hr at 37°C. **b** Growth inhibition by high concentrations of MA in an iron-limiting minimal medium. Wild-type and *ΔfeuA* strains were streaked on SMM plates with paper disc containing MA, and incubated for 30-42 hr at 37°C. **c** Growth of *B. subtilis* mediated by an iron utilization with MA. *ΔfecC* mutant were streaked on SMM plates with or without various concentrations of MA, as indicated, and incubated for 30-42 hr at 37°C. **d** Growth of an *mbl* mutant in a minimal medium. Wild-type (168CA) and *Δmbl* (YK2638) strains were streaked on SMM plates with or without 10 mM Mg^{2+}, 10 μg/ml MA or MC, and incubated for 30-42 hr at 37°C. **e** Effects of iron availability on *mbl* mutant growth in a minimal medium. *mbl* mutant strains (YK2638; *Δmbl*, YK2771; *Δmbl ΔfecC*) were streaked on SMM plates with or without added 200 μM ferric chloride (Fe^{3+}), and incubated for 30-42 hr at 37°C.

The figures are representative of at least three independent experiments.

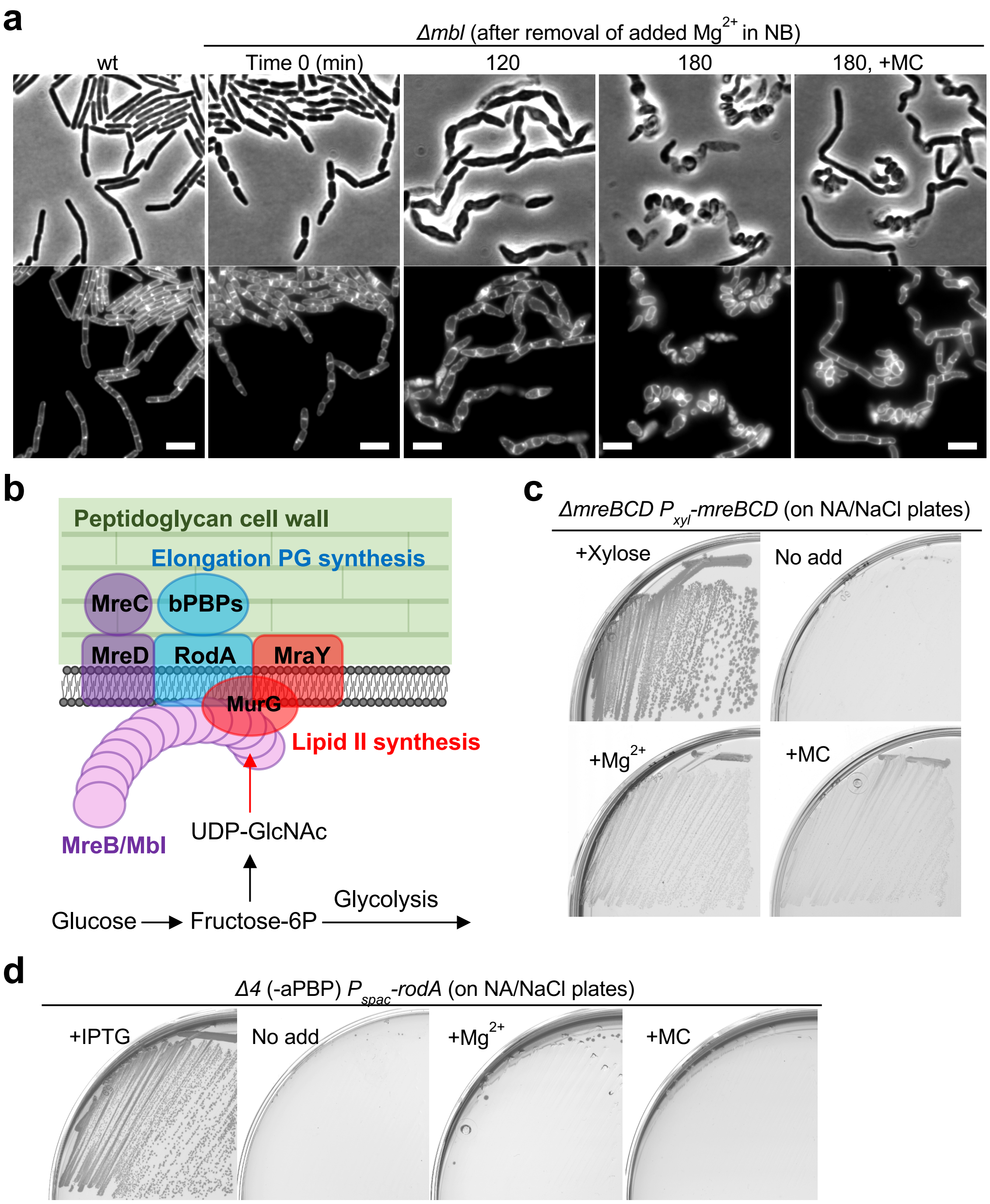
a
Δmbl (after removal of added Mg2+ in NB)
wt
Time 0 (min)
120
180
180, +MC
b
Peptidoglycan cell wall
Elongation PG synthesis
MreC
bPBPs
MreD
RodA
MraY
MurG
Lipid II synthesis
MreB/Mbl
UDP-GlcNAc
Glucose
Fructose-6P
Glycolysis
c
ΔmreBCD Pxyl-mreBCD (on NA/NaCl plates)
+Xylose
No add
+Mg2+
+MC
d
Δ4 (-aPBP) Pspac-rodA (on NA/NaCl plates)
+IPTG
No add
+Mg2+
+MC

Supplementary Fig. 2. **Growth rescue of elongation mutants by MC. a** Suppression of a toxic effect in an *mbl* mutant by added Mg^{2+} or MC. *B. subtilis* wild-type strain (168CA) was cultured in NB, and phase contrast micrograph and the corresponding membrane staining image for the exponentially growing cells were captured. An *mbl* mutant strain was cultured in NB containing 10 mM Mg^{2+} (Time 0), and the cells were diluted into fresh NB (no added Mg^{2+}) with or without 10 µg/ml MC and incubated for 2-3 hr. Phase contrast micrographs and the corresponding membrane staining images were captured after the dilution. Scale bars represent 5 µm. **b** Schematic representation of key factors for PG synthesis during cell wall elongation in *B. subtilis*. **c** Growth rescue by MC in a strain lacking *mreB*, *mreC* and *mreD* genes (*ΔmreBCD*). YK1875 (*ΔmreBCD amyE*::P_{xyl}-*mreBCD*) was streaked on NA plates containing 100 mM NaCl (for osmoprotection), with or without 0.5 xylose, 10 mM Mg^{2+} or 40 µg/ml MC, and incubated for 24 hr at 37°C. **d** aPBP-dependent growth rescue by MC in a *rodA* mutant. YK2242 (*Δ4* P_{spac}-*rodA*) was streaked on NA plates containing 100 mM NaCl (for osmoprotection), with or without 0.1 mM IPTG, 10 mM Mg^{2+} or 40 µg/ml MC, and incubated for 24 hr at 37°C.

The figures are representative of three independent experiments.

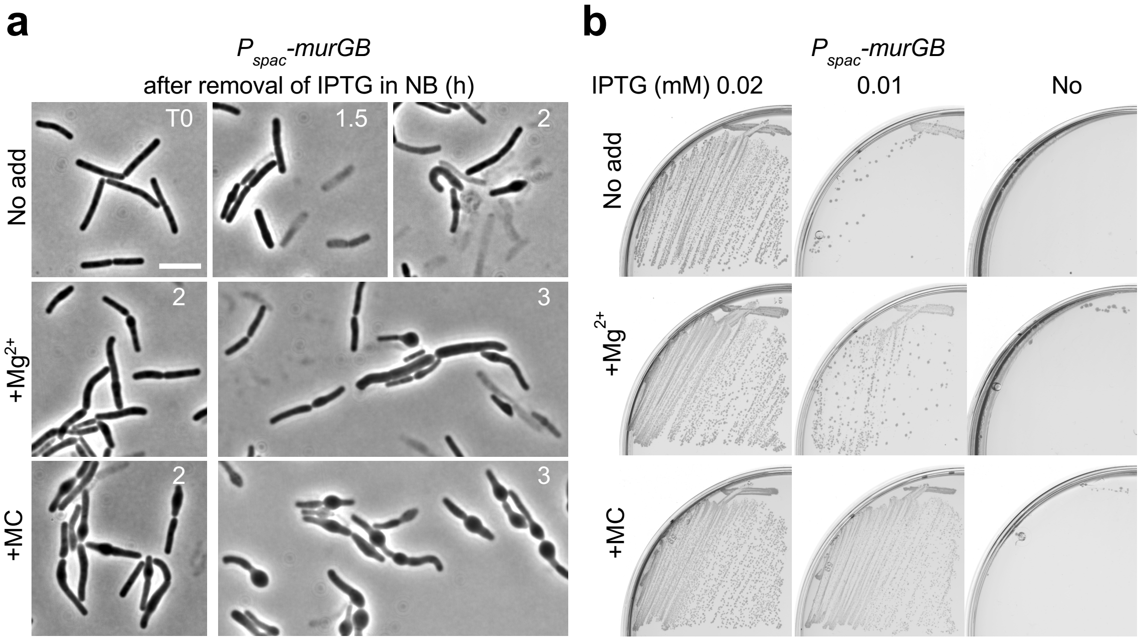

Supplementary Fig. 3. **Growth rescue by MC during PG precursor depletion. a** Effects of MC and Mg^{2+} on cell morphology and lysis during *murGB* repression. Phase contrast micrographs of *B. subtilis* cells in the course of carrying out the growth curves shown in Figure 3A. *B. subtilis* strain YK1540 (P_{spac}-*murG-murB*) was cultured in NB containing 0.1 mM IPTG at 37°C (T0, hr). The exponentially growing cells were diluted into fresh NB (without IPTG) with 10 µg/ml MC or 10 mM Mg^{2+} and incubated for 1.5-3 hr. Scale bars represent 5 µm. **b** Growth rescue by MC during *murGB* repression. The YK1540 was streaked on NA plates containing IPTG, with or without 10 mM Mg^{2+} or 10 µg/ml MC, as indicated.

The figures are representative of at least three independent experiments.

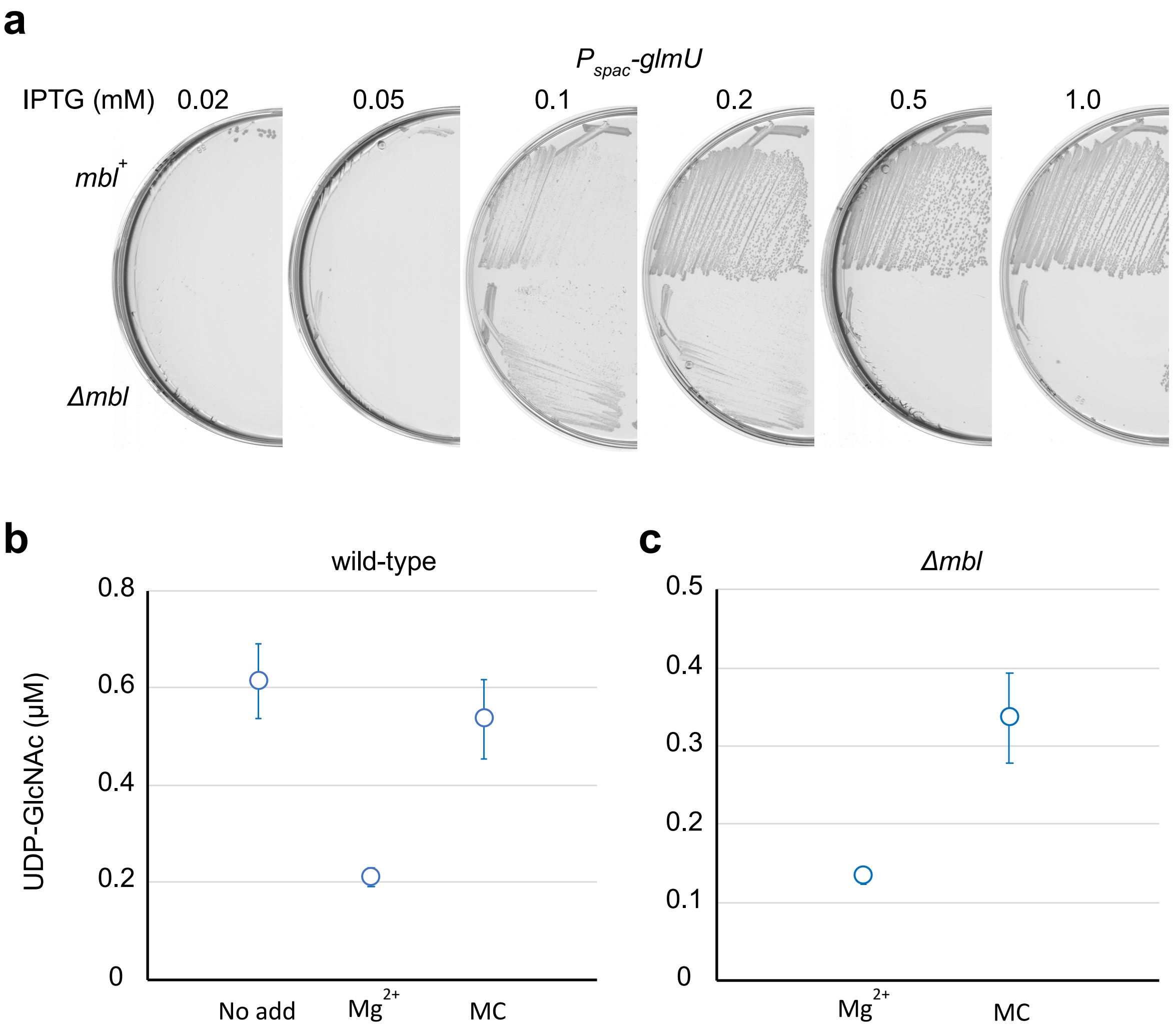

Supplementary Fig. 4. **Growth rescue of *mbl* mutant by Mg^{2+} associates with UDP-GlcNAc synthesis.** **a** Growth rescue of an *mbl* mutant by reducing UDP-GlcNAc synthesis. *B. subtilis* strains YK1538 (*P_{spac}-glmU*) and YK2687 (*Δmbl P_{spac}-glmU*) were streaked on NA plates containing various concentrations of IPTG, as indicated, and incubated for 18 hr at 37°C. The figures are representative of three independent experiments. **b** Effect of Mg^{2+} on intracellular UDP-GlcNAc concentrations. Wild-type strain (168CA) was cultured in LB with or without 10 mM Mg^{2+} or 10 µg/ml MC at 37°C to an OD_{600}=0.5. Cells were harvested from 7 ml of culture by centrifugation, washed and used for obtaining samples for measurements of UDP-GlcNAc levels. The means were obtaind from three independent experiments. Error bars indicate standard deviation (SD). Source data are provided as a Source Data file. **c** Mg^{2+}-dependent reduction of UDP-GlcNAc levels in an *mbl* mutant. YK2638 (*Δmbl*) was cultured in LB containing 10 mM Mg^{2+} and diluted into fresh LB with 10 mM Mg^{2+} or 10 µg/ml MC. The cells were incubated at 37°C to OD_{600}=0.5 for measurements of UDP-GlcNAc levels. The means were obtained from three independent experiments. Error bars indicate SD. Source data are provided as a Source Data file.

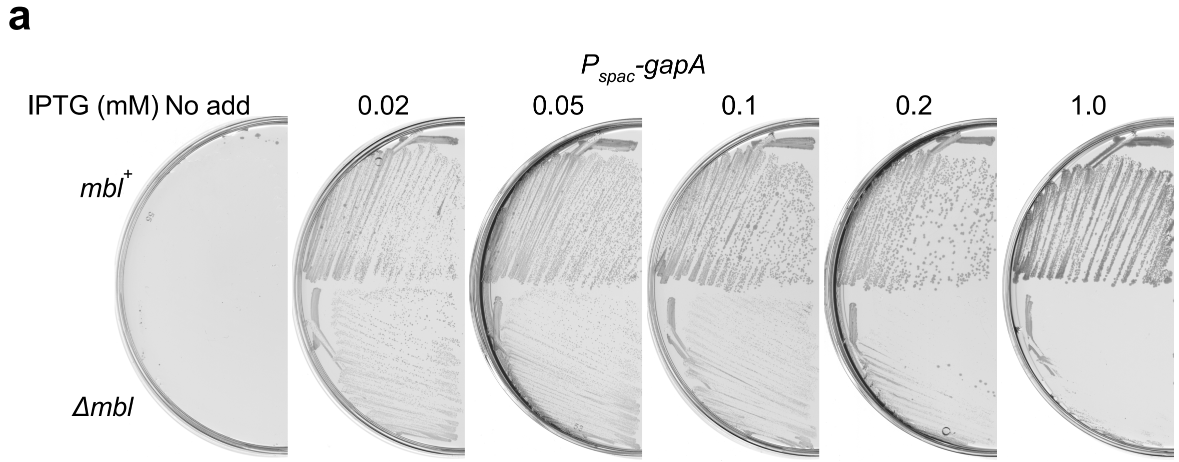

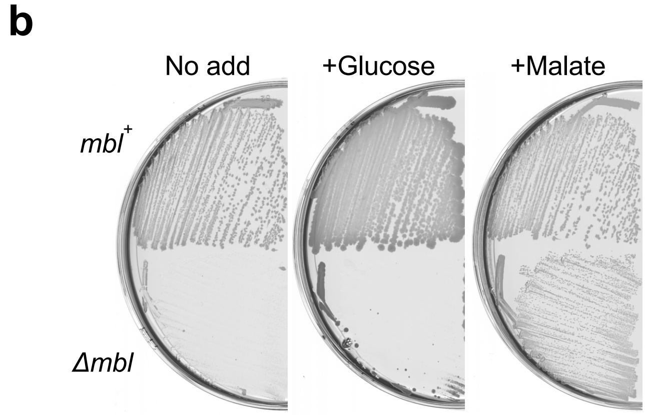

Supplementary Fig. 5. ***mbl* mutant lethality associates with glycolysis.** **a** Effects of *gapA* expression levels on *mbl* mutant growth. *B. subtilis* strains YK1567 (P_{spac}-*gapA*) and YK2711 (*Δmbl* P_{spac}-*gapA*) were streaked on NA plates containing various concentrations of IPTG, as indicated, and incubated for 18 hr at 37°C. **b** Effects of glycolytic or gluconeogenic carbon source on growth of an *mbl* mutant. Wild-type (168CA) and *Δmbl* (YK2638) strains were streaked on NA with or without 0.2% glucose or malate and incubated for 18 hr at 37°C.

The figures are representative of at least three independent experiments.

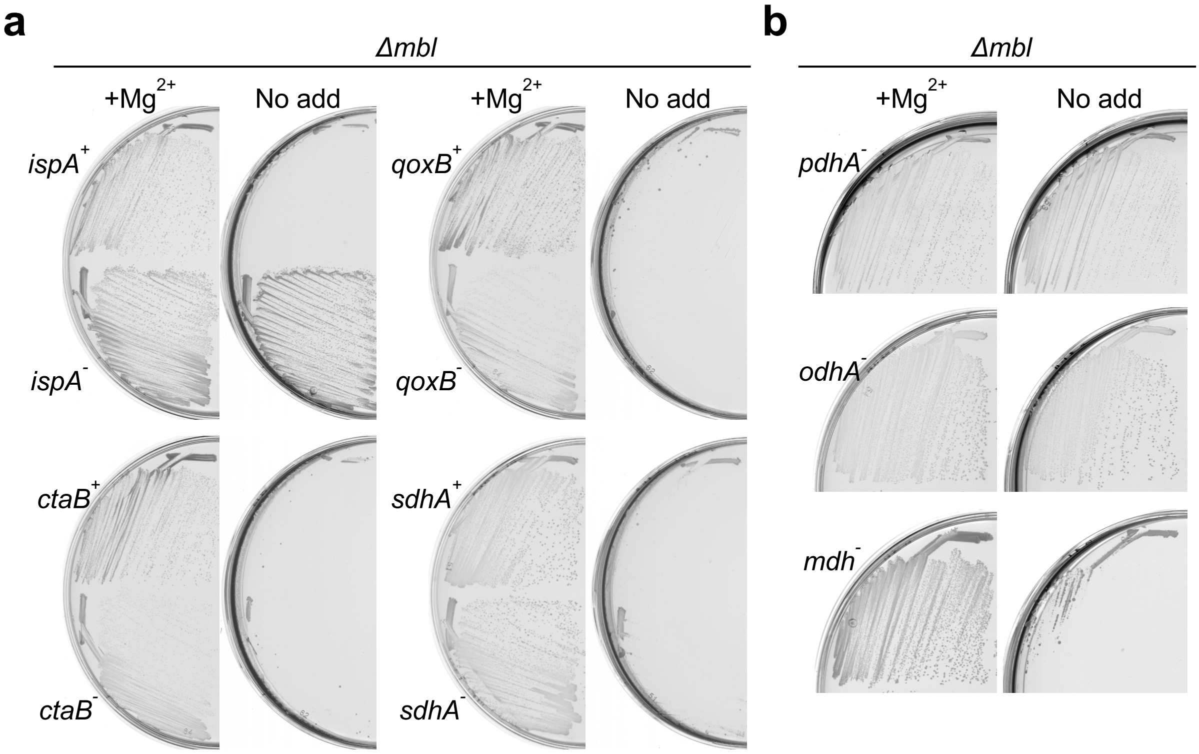

Supplementary Fig. 6. **Effects of respiratory chain enzymes and NADH production on *mbl* mutant lethality. a** *B*. *subtilis* strains YK2638 (*Δmbl*), YK2694 (*ispA*- *Δmbl*), YK2696 (*ΔqoxB Δmbl*), YK2697 (*ΔctaB Δmbl*) and YK2718 (*ΔsdhA Δmbl*) were streaked on NA plates with or without 10 mM Mg^{2+} and incubated for 18 hr at 37°C. **b** YK2732 (P_{spac}-*pdhA Δmbl*), YK2716 (*ΔodhA Δmbl*) and YK2720 (*Δmdh Δmbl*) were streaked on NA plates with or without 10 mM Mg^{2+} and incubated for 18 hr at 37°C.

The figures are representative of at least three independent experiments.

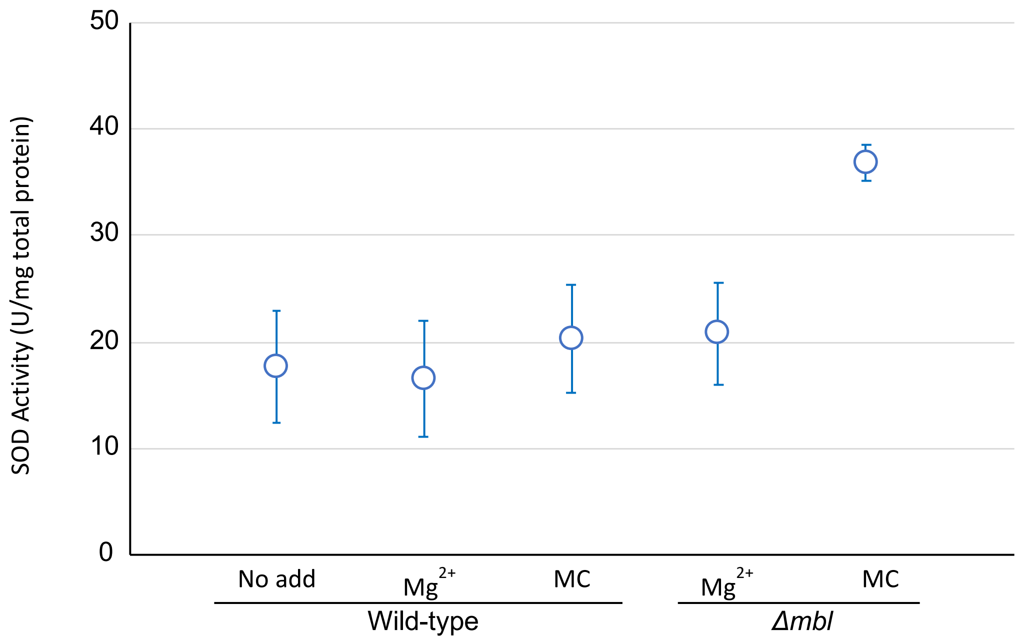

Supplementary Fig. 7. **Enhancement of SOD activity in an *mbl* mutant and its suppression by Mg^{2+}.** *B. subtilis* strains 168CA (wild-type) and YK2638 (Δ*mbl*) were cultured with or without 20 mM Mg^{2+} or 10 µg/ml MC at 37°C to OD_{600}=0.6. SOD activity was measured in lysates of the cells. The means were obtained from three independent experiments. Error bars indicate SD. Source data are provided as a Source Data file.

Supplementary Table 1. Bacterial strains

Strains	Genotypes	References
B. subtilis		
Marburg (NCIB3610)	wild-type	Lab. stock
168CA	*trpC2* (wild-type)	Lab. stock
BS115	*trpC2 ΩspoVD::cat-*P_{xyl}*-murE*	[1]
LR2	*trpC2 ΩspoVD::cat-*P_{xyl}*-murE* $ispA^-$	[2]
YK1395	*trpC2* $ispA^-$ (*xseB::Tn-kan*)	[2]
YK1450	*trpC2 ΩhepS::erm-*P_{spac}*-hepS*	[3]
YK1538	*trpC2 ΩglmU::erm-*P_{spac}*-glmU*	This work
YK1540	*trpC2 ΩmurG::erm-*P_{spac}*-murG-murB*	This work
YK1567	*trpC2 ΩgapA::erm-*P_{spac}*-gapA*	[4]
YK1714	*trpC2 ndh::Tn-kan*	[3]
YK1875	*trpc2 Ωneo-ΔmreB-mreC-mreD amyE::*P_{xyl}*-mreB-mreC-mreD spc*	[5]
YK2242	*trpC2 Δ4* P_{spac}*-rodA kan*	[6]
YK2245	*trpC2* P_{spac}*-rodA kan*	[6]
YK2265	*trpC2 aprE::*P_{rpsD}*-mcherry spc*	[3]
YK2625	*trpC2 Δmbl::cat ΩhepS::erm-*P_{spac}*-hepS*	[3]
YK2638	*trpC2 Δmbl::cat*	[7]
YK2646	*trpC2 Δmbl::cat ndh::Tn-kan*	[3]
YK2665	*trpC2 Δmbl::cat ΩmurG::erm-*P_{spac}*-murG-murB*	This work
YK2687	*trpC2 Δmbl::cat ΩglmU::erm-*P_{spac}*-glmU*	This work
YK2689	*trpC2 ΩmurG::erm-*P_{spac}*-murG-murB ndh::Tn-kan*	This work
YK2694	*trpC2 Δmbl::cat* $ispA^-$ (*xseB::Tn-kan*)	[2]
YK2696	*trpC2 Δmbl::cat qoxB::Tn-kan*	[3]
YK2697	*trpC2 Δmbl::cat ctaB::Tn-kan*	[3]
YK2700	*trpC2 Δmbl::cat amyE::*P_{xyl}*-murB-spc*	This work
YK2701	*trpC2 Δmbl::cat amyE::*P_{xyl}*-murG-spc*	This work
YK2711	*trpC2 Δmbl::cat ΩgapA::erm-*P_{spac}*-gapA*	This work
YK2716	*trpC2 Δmbl::cat ΩodhA::erm ΔodhA*	This work
YK2718	*trpC2 Δmbl::cat ΩsdhA::erm ΔsdhA*	This work
YK2720	*trpC2 Δmbl::cat Ωmdh::erm Δmdh*	This work
YK2732	*trpC2 Δmbl::cat ΩpdhA::erm-*P_{spac}*-pdhA*	This work
YK2739	*trpC2 ΔefeU::kan*	[8]
YK2740	*trpC2 ΔfecC::kan*	[8]
YK2741	*trpC2 ΔfeuA::kan*	[8]
YK2771	*trpC2 Δmbl::cat ΔfecC::kan*	[8]
E. coli		
BW25113	$lacI^+ rrnB_{T14}$ $\Delta lacZ_{WJ16}$ *hsdR*514 $\Delta araBAD_{AH33}$ $\Delta rhaBAD_{LD78}$ *rph-1* *Δ(araB–D)567 Δ(rhaD–B)568 ΔlacZ4787*(*::rrnB-3*) *hsdR514 rph-1*	Lab. stock

Supplementary Table 2. Primers

Primers	Sequences
pMSD-glmU-F	GGGGAATTCTTCTATGGATAAAAGGGATATTG
pM-glmU-R	GGGGGATCCAAGCGGCGTATCTCCGCAA
pMSD-murGB-F	GGGGAATTCTTATCGTTATGTTATAGAGAAACT
pM-murGB-R	GGGGGATCCCCGGCCCACATACATAACC
pMSD-pdhA-F	GAAGAATTCAATGCACGTCTAATGACTG
pM-pdhA-R	GGAGGATCCCGCGAAATGCGTTGCAATC
pM-sdhA-F	GAAGAATTCTAAAGCAGCGGAATCAGG
pM-sdhA-R	GGAGGATCCTTA TCGTCGCACCAGCATAAG
pM-mdh-F	GAAGAATTCGAGCTGGCAGACGTTGTTC
pM-mdh-R	GGAGGATCC TTACCTGACTGGCCGATTACAC
MK116	GAGGAAGCGGAAGAATGAAG
MK117	TTCGGTAAGTCCCGTCTAGC
MK532	CCCAGTCTAAGATGCATTTTATGTCATATTG
MK533	GACATAAAATGCATCTTAGACTGGGGGAAAAAAGAAATGC
MK534	GCTCTAGAACTAGAATTCCTATTTTTTTAATTCCTCGAGTACG
MK535	CGTACTCGAGGAATTAAAAAAATAGGAATTCTAGTTCTAGAGC
MK536	CTCCGCATTCCATAGATGCATTTTATGTCATATTG
MK537	GACATAAAATGCATCTATGGAATGCGGAGGTTTAC
MK538	CTCTAGAACTAGAATTCTCAGCGATTTCCGCCGATG
MK539	CATCGGCGGAAATCGCTGAGAATTCTAGTTCTAGAGC

SUPPLEMENTARY REFERENCES

1 Leaver, M. Dominguez-Cuevas, P. Coxhead, J. M. Daniel, R. A. & Errington, J. Life without a wall or division machine in *Bacillus subtilis*. *Nature* **457**, 849-853 (2009). doi.org:10.1038/nature07742

2 Mercier, R. Kawai, Y. & Errington, J. Excess membrane synthesis drives a primitive mode of cell proliferation. *Cell* **152**, 997-1007 (2013). doi.org:10.1016/j.cell.2013.01.043

3 Kawai, Y. *et al.* Cell growth of wall-free L-form bacteria is limited by oxidative damage. *Curr Biol* **25**, 1613-1618 (2015). doi.org:10.1016/j.cub.2015.04.031

4 Kawai, Y. *et al.* Crucial role for central carbon metabolism in the bacterial L-form switch and killing by β-lactam antibiotics. *Nat Microbiol.* (2019). doi.org:10.1038/s41564-019-0497-3

5 Leaver, M. & Errington, J. Roles for MreC and MreD proteins in helical growth of the cylindrical cell wall in *Bacillus subtilis*. *Mol Microbiol.* **57**, 1196-1209 (2005). doi.org:10.1111/j.1365-2958.2005.04736.x

6 Emami, K. *et al.* RodA as the missing glycosyltransferase in *Bacillus subtilis* and antibiotic discovery for the peptidoglycan polymerase pathway. *Nat Microbiol* **2**, 16253 (2017). doi.org:10.1038/nmicrobiol.2016.253

7 Schirner, K. & Errington, J. The cell wall regulator {sigma}I specifically suppresses the lethal phenotype of *mbl* mutants in *Bacillus subtilis*. *J Bacteriol* **191**, 1404-1413 (2009). doi.org:10.1128/JB.01497-08

8 Koo, B. M. *et al.* Construction and Analysis of Two Genome-Scale Deletion Libraries for *Bacillus subtilis*. *Cell Syst* **4**, 291-305 e297 (2017). doi.org:10.1016/j.cels.2016.12.013
